# Effects of a Dietary Supplement on Systemic Aging Related Biomarkers and Skin Aging Outcomes in Middle‐Aged Women: In Vitro and Clinical Studies

**DOI:** 10.1111/jocd.71175

**Published:** 2026-09-06

**Authors:** Jie‐Hua Chen, Liugang Ding, Pingping Lv, Wenzhi Li, Meiling Tai, Ran Yin, Yi Yu, Yunru Huang, Jinluan Zheng, Erman Wang, Huan Liu, Jiaojiao Fu, Hongjue Wang, Ruopan Huang, Jun Ma

**Affiliations:** ^1^ China‐UK Joint Laboratory on Health and Ageing Research Guangzhou China; ^2^ Infinitus (China) Company Ltd. Guangzhou China; ^3^ Landproof Testing Technology Co., Ltd. Guangzhou China; ^4^ South China Biochip Research Center Guangzhou China; ^5^ RayBiotech, Inc. Guangzhou China; ^6^ Guangdong Provincial Hospital of Chinese Medicine Guangzhou China

**Keywords:** DNA methylation‐based biological age, elasticity, PPQ, skin aging, systemic aging, wrinkles

## Abstract

**Background:**

Interest in dietary supplements targeting both skin and biological aging biomarkers is rapidly growing, yet existing studies lack rigorous clinical validation of combined oral–topical regimens and concurrent assessment of skin and systemic biomarkers.

**Objective:**

This study aimed to evaluate skin aging‐related outcomes together with selected systemic biomarkers associated with aging biology.

**Methods:**

Human skin fibroblasts were treated with Oriherb FuHuoLvShi (FHLS), a revitalizing dietary supplement adapted from Traditional Chinese Medicine classical formula *QiongYu* Paste, together with PQQ, astaxanthin, and apple polyphenols. Senescence‐associated parameters including β‐galactosidase (SA‐β‐gal), γ‐H2AX foci, reactive oxygen species (ROS), Type I and III collagen expression were measured. Two 12‐week clinical trials were conducted in middle‐aged women: (1) an open‐label FHLS‐Only trial investigating DNA methylation‐based biological age, pro‐inflammatory cytokines, NAD levels, and gene expressions of sirtuins in blood samples, in addition to skin aging phenotypes; (2) a randomized parallel‐controlled combination trial comparing topical plus oral FHLS with topical application alone.

**Results:**

FHLS significantly reduced SA‐β‐gal, γ‐H2AX foci and ROS while increasing Type I and III collagen expression in vitro. After 12 weeks, FHLS improved skin hydration (+62.46%), TEWL (−18.69%), radiance (+17.19%), elasticity (+15.16%), firmness (−14.85%), dermal density (+32.29%), and facial wrinkle severity (*p* < 0.001). Systemically, FHLS significantly increased NAD^+^, total NAD levels and the expression of sirtuin genes (SIRT 1, SIRT 3 and SIRT 6), while decreasing levels of pro‐inflammatory cytokines IL‐6, IL‐2 and TNF‐α in blood. Notably, DNA methylation age was reduced by an average of 0.84 years after the 12‐week intervention. Though this result shows a promising effect of the intervention strategy, it should be interpreted with caution and warrants further investigation. Furthermore, combined oral–topical treatment showed superior efficacy on skin aging parameters compared to topicals alone.

**Conclusion:**

FHLS was associated with improvements in multiple skin aging‐related outcomes and changes in selected systemic aging biomarkers. These findings support further investigation into larger placebo‐controlled studies.

## 1 Introduction

Aging does not occur through a single pathway but reflects the combined effect of several interconnected biological processes, including genomic instability, epigenetic changes, mitochondrial dysfunction, cellular senescence, and chronic low‐grade inflammation [1, 2]. Over time, these processes begin to disrupt normal tissue homeostasis across different organs. The skin, being the largest and most visible organ, is affected in a particularly noticeable way and can also reflect broader physiological changes at the systemic level [3]. In clinical terms, aging skin typically shows reduced hydration and a weakened barrier, along with a gradual loss of elasticity and firmness. Dermal thinning and wrinkle formation are also commonly observed [4]. These features are closely related to changes in the dermal extracellular matrix, especially the breakdown and disorganization of collagen and elastin [5, 6].

Skin aging is not solely a local process, as systemic biological aging also contributes to structural changes in the skin and a reduced capacity for repair. Senescent cells, through their senescence‐associated secretory phenotypes (SASPs), can promote chronic inflammation, often described as “inflammaging,” and accelerate ECM breakdown via cytokines and proteases [7, 8]. At the same time, DNA methylation‐based measures of biological age are increasingly used as integrative biomarkers, reflecting the cumulative burden of aging and, in some cases, linking these systemic changes to skin health [9].

Current anti‐aging approaches typically fall into three categories: topical formulations, aesthetic procedures, and oral supplements. Topical agents act primarily on superficial layers of skin and often exhibit limited efficacy due to poor penetration [10]. Aesthetic procedures, by contrast, may provide temporary structural correction, but they tend to be costly and impose significant procedural burdens [11]. Oral nutritional approaches may exert systemic effects on aging‐related pathways. However, existing clinical evidence remains heterogeneous, and few studies have examined combined oral–topical regimens with systemic aging biomarkers.

To address these limitations, a multi‐level evaluation was designed. We first aimed to determine whether a novel dietary supplement could influence both skin aging phenotypes and systemic aging biology, and whether combining oral and topical approaches would provide additional benefit compared with topical treatment alone. At the cellular level, the evaluation focused on markers of cellular senescence, oxidative stress, DNA damage, and collagen production in human skin fibroblasts (HSFs). Building on this, a 12‐week clinical study was conducted to track changes in skin physiological parameters together with circulating inflammatory cytokines, gene expression of sirtuins, and DNA methylation‐based biological age. A randomized parallel‐controlled trial was also included to examine whether the combined oral–topical regimen offered added benefit over topical treatment alone. Overall, this design was intended to link findings across different levels, from cellular mechanisms to clinical skin outcomes and systemic biomarkers, rather than treating them as separate observations.

## 2 Materials and Methods

### 2.1 Study Design and Sub‐Studies

**FIGURE 1:**
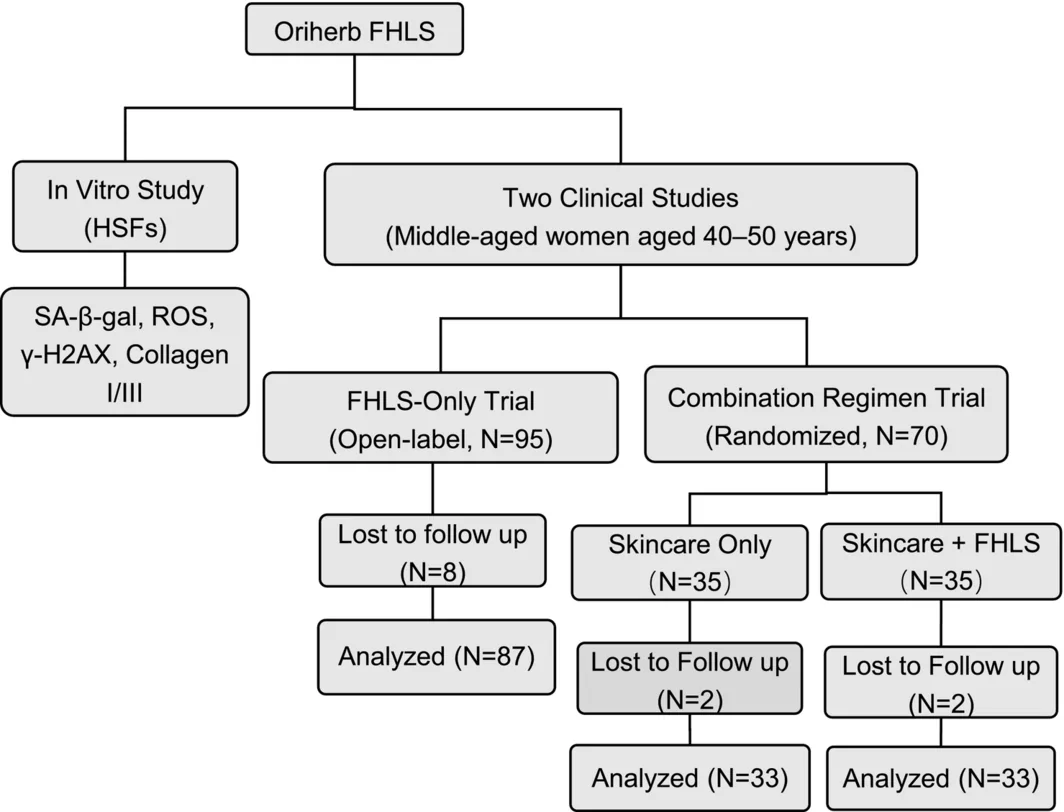
Study design and sub‐studies.

This work comprised (i) an in vitro mechanistic study in HSFs and (ii) two clinical studies in middle‐aged women (Figure 1). Clinical Study 1, Oriherb FuHuoLvShi (FHLS)‐Only Trial, was an open‐label, single‐arm 12‐week supplementation study designed to evaluate changes in objective skin parameters over time and to explore selected systemic biomarkers measured at baseline and Week 12. Clinical Study 2, Combination Regimen Trial was a randomized, parallel‐controlled 12‐week study comparing a standardized topical skincare regimen alone versus the same topical regimen in combination with oral dietary supplements (FHLS; see Section 2.2).

Primary endpoint for FHLS‐Only Trial was changes in DNA methylation‐based biological age, whereas secondary endpoints included 12‐week changes in facial wrinkle severity, skin elasticity (R2), transepidermal water loss (TEWL), pro‐inflammatory cytokines, NAD levels, and gene expressions of sirtuins in blood samples, in addition to skin aging phenotypes. The primary endpoint of the combination regimen trial was between‐groups comparisons of crow's feet wrinkles. Secondary endpoints included forehead lines, nasolabial folds, skin elasticity (R2), and TEWL. In this trial, participants were randomly assigned in a 1:1 ratio to the skincare‐alone group or the skincare + FHLS group using a pre‐specified randomization procedure.

Efficacy analyses were conducted in the per‐protocol population (Study 1: *N* = 87; Study 2: *N* = 66), defined a priori as participants who completed the intervention without major protocol deviations and had endpoint data available at the prespecified assessment time point. Participants who withdrew or were lost to follow‐up before the endpoint visit were excluded. No missing data were present for the analyzed endpoints in the per‐protocol population.

### 2.2 Study Product

The study product, Oriherb FHLS, was provided by Infinitus (China) Company Ltd. as a powdered dietary supplement packaged in single‐dose sachets for once‐a‐day oral administration. The formulation consisted of an adapted Traditional Chinese Medicine classical formula *Qiongyu* Paste containing red ginseng extract, *Cordyceps militaris* extract, black goji berry extract, *Poria cocos* extract, *Dendrobium officinale* extract, and *Rehmannia radix* extract. The formulation also contained pyrroloquinoline quinone (PQQ), astaxanthin, and apple polyphenols. All botanical raw materials were authenticated prior to extraction, and standardized extracts were prepared to ensure batch‐to‐batch consistency. High‐performance liquid chromatography (HPLC) and polysaccharide data of FHLS are provided in the Supporting Information.

### 2.3 Liquid Chromatography‐High Resolution Mass Spectrometry (LC‐HRMS) Analysis of FHLS

The compounds in FHLS were profiled by LC‐HRMS. For sample preparation, a certain amount of FHLS was fully dissolved in methanol and extracted ultrasonically at room temperature for 30 min. Then, the extract was centrifuged at 4696 g for 10 min, and the resulting supernatant was passed through a 0.22 μm membrane filter before injection. Chromatographic separation was achieved on a C18 reversed‐phase column (2.1 × 100 mm, 1.9 μm) with a mobile phase consisting of 0.1% formic acid in water (solvent A) and 0.1% formic acid in acetonitrile (solvent B). Gradient elution was performed with solvent B maintained at 5% during 0–2 min, linearly increased from 5% to 95% over 2–42 min, held at 95% from 42 to 47 min, rapidly decreased from 95% to 5% over 47–47.1 min, and then equilibrated at 5% from 47.1 to 50 min. The flow rate was set at 0.3 mL/min, with the column temperature maintained at 40°C and the injection volume fixed at 3 μL. Mass spectrometric acquisition was performed using an electrospray ionization source with a spray voltage of 3.5 kV, capillary temperature of 320°C, sheath gas flow of 35 arb, and auxiliary gas flow of 10 arb. Data were collected in Full MS‐ddMS^2^ mode over an *m/z* range of 100–1500, with stepped normalized collision energies of 20, 40, and 60. The resulting LC‐HRMS datasets were processed for putative identification of FHLS‐derived chemical constituents.

### 2.4 In Vitro Anti‐Senescence Assays in HSFs

#### 2.4.1 Cell Culture and Treatment Preparation

HSFs were purchased from Laibo Cosmeceutical Technology (Shanghai) Co. Ltd., and used for anti‐senescence assays. The cells were cultured in standard medium supplemented with 10% (v/v) fetal bovine serum and 1% penicillin–streptomycin, and maintained at 37°C in a humidified incubator with 5% CO_2_. To ensure experimental consistency and reproducibility, cells at passages 4–8 were used in all assays. Cell viability was assessed using the CCK‐8 assay, which showed that FHLS exhibited no significant cytotoxicity at concentrations up to 10 mg/mL. Based on this result, 2 mg/mL was selected as a safe, sub‐cytotoxic concentration for the subsequent anti‐senescence experiments.

#### 2.4.2 SA‐β‐Galactosidase (SA‐β‐Gal) Activity Assay

Following induction of senescence with 200 mM D‐galactose, HSFs were treated with FHLS or 1 mM N‐acetylcysteine (NAC, positive control), along with model and negative control groups. Cellular senescence was evaluated by measuring SA‐β‐galactosidase activity using a senescence detection kit (Beyotime Biotechnology, China) according to the manufacturer's instructions. After performing the staining per the kit protocol and DAPI (Beyotime Biotechnology, China) counterstaining, the percentage of SA‐β‐galactosidase positive (blue‐stained) cells was quantified from randomly selected microscopic fields in each well.

#### 2.4.3 Measurement of Intracellular Reactive Oxygen Species (ROS)

Intracellular ROS levels were measured using the fluorescent probe DCFH‐DA. HSFs were pretreated for 4 h as follows: (1) negative control (medium alone); (2) senescence model (medium alone); (3) positive control (1 mM NAC); and (4) FHLS treatment (2 mg/mL). Following pretreatment, all groups except the negative control were exposed to 100 μM H_2_O_2_ for 1 h to induce ROS generation. Cells were incubated with DCFH‐DA (1:2000 dilution) at 37°C for 20 min according to the manufacturer's instructions (ROS Assay Kit, Solarbio, China). After washing, fluorescence images were captured using a fluorescence microscope (FITC channel), and the mean fluorescence intensity per field was quantified for analysis.

#### 2.4.4 Immunofluorescence Detection of γ‐H2AX Foci

DNA damage was assessed by immunofluorescence detection of γ‐H2AX foci using the same pretreatment groups and H_2_O_2_ exposure protocol described for the intracellular ROS assay. Following oxidative stress induction, the culture medium was removed, and cells were fixed and subjected to γ‐H2AX immunostaining using a commercial assay kit (Beyotime Biotechnology, China) according to the manufacturer's instructions. After incubation with anti‐γ‐H2AX primary antibody and the corresponding Alexa Fluor 488‐conjugated secondary antibody, nuclei were counterstained with DAPI for 5 min. Fluorescence images were acquired using a microscope (MI52‐N, Mshot, China), and the mean γ‐H2AX fluorescence intensity per nucleus was quantified for analysis.

#### 2.4.5 Quantification of Type I and Type III Collagen

Type I and III collagen were quantified by enzyme‐linked immunosorbent assay (ELISA). Cells were treated for 24 h with 2 mg/mL FHLS, 100 ng/mL TGF‐β1 (positive control), or medium alone (negative control). Subsequently, culture supernatants were collected and analyzed using commercial ELISA kits for human Type I Collagen and Type III Collagen (Mlbio, China). Absorbance was measured at 450 nm.

### 2.5 Clinical Studies

#### 2.5.1 Ethics, Participants, and General Procedures

The clinical study was conducted in accordance with the ethical principles of the Declaration of Helsinki. The study protocol and procedures were approved by the Ethics Committee, and written informed consent was obtained from all participants participating in the study. Safety and tolerability monitoring was performed via dermatologist examinations, interviews, and daily adverse event questionnaires.

Eligible participants were healthy Chinese female volunteers aged 40–50 years who met the following inclusion criteria: (1) > 7 days since last menstrual period; (2) baseline TEWL > 10 g/m^2^/h on the cheek area; (3) ability to understand the study‐related information and provide written informed consent; (4) willingness to refrain from using any other products or treatments with similar efficacy (e.g., collagen supplements) during the study period; and (5) no intake of anti‐aging relevant supplements in the past 2 months. Individuals were excluded if they met any of the following conditions: (1) pregnancy or lactation; (2) history of food allergies or food intolerances; (3) presence of acute or chronic skin diseases, or visible skin conditions (e.g., sunburn, hyperpigmentation) that could interfere with assessments; (4) uncontrolled chronic or endocrine diseases; (5) use of medications affecting skin condition or receipt of aesthetic procedures on the test area within the past 6 months; (6) participation in another clinical trial within the past 3 months; and (7) any other medical or personal condition that could affect the study in the investigator's judgment. All participants exhibited clinical signs of skin aging, including dryness, reduced elasticity and radiance, and visible facial wrinkles.

#### 2.5.2 Clinical Study 1: FHLS‐Only Open‐Label Supplementation

An open‐label, 12‐week clinical study was conducted to evaluate the anti‐aging efficacy of the study product. A total of 95 female participants consumed one sachet (5 g) of the investigational product daily. DNA methylation‐based biological age was analyzed at baseline and 12 weeks. In addition, skin parameters, including skin hydration, TEWL, radiance, elasticity (R2), and firmness (F4), were measured at baseline (before intervention), 2 weeks (2 weeks after intervention), 4 weeks (4 weeks after intervention), and 12 weeks (12 weeks after intervention). Dermal density and wrinkle severity (assessed by the number and length of cheek fine lines, forehead wrinkles, crow's feet wrinkles, nasolabial folds, glabellar lines, under‐eye wrinkles, tear trough, and wrinkles at lip corners) were assessed at baseline, 4 and 12 weeks. Blood samples were collected at baseline and at Week 12 for the analysis of five pro‐inflammatory cytokines (IFN‐γ, IL‐6, IL‐2, TNF‐α, and hsCRP), mRNA expression levels of sirtuins genes (SIRT1, SIRT3, and SIRT6), NAD^+^, and total NAD.

#### 2.5.3 Clinical Study 2: Combination Regimen Randomized Parallel‐Controlled Trial

To evaluate anti skin‐aging effects of combining the oral dietary supplement with topical skincare, an 84‐day, randomized, parallel‐controlled trial was conducted. Seventy female participants were randomized into two groups: (1) Skincare group (*N* = 35): followed a standardized twice daily skincare regimen. (2) Skincare + FHLS group (*N* = 35): followed the identical skincare regimen plus the dietary supplement taken once daily. The topical skincare regimen consisted of an essence and a face cream (provided by Infinitus (China) Company Ltd.). Crow's feet wrinkles were assessed at baseline and after 2, 4, and 12 weeks of intervention. Meanwhile, the same skin parameters evaluated in the open‐label study, namely skin hydration, TEWL, radiance, elasticity, firmness, dermal density, and facial wrinkle severity, were assessed at baseline and after 2, 4, and 12 weeks.

#### 2.5.4 Skin Instrumental Assessments

Skin parameters were evaluated following advanced dermatological instruments and standardized protocols under controlled environmental conditions (21°C ± 1°C, 50% ± 5% humidity). Cheek hydration was assessed with a Corneometer CM 825 (Courage and Khazaka, Germany), measuring stratum corneum capacitance. TEWL was evaluated using a Tewameter TM 300 (Courage and Khazaka, Germany) based on the open chamber principle to determine the skin's barrier function. Skin radiance was measured using the SkinGlossMeter SG2001, mean ± standard deviation (SD) values determined from triplicate testing at cheek were used for data analysis. Skin elasticity and firmness were evaluated on the cheek using the Cutometer Dual MPA 580 (Courage and Khazaka, Germany), yielding metrics for R2 (total elasticity) and F4 (firmness). Higher R2 values indicate improved elasticity, while lower F4 values reflect better firmness. A non‐invasive, skin three‐dimensional imaging system (Primos CR, Canfield Co. USA) was used to measure the number and length of facial wrinkles (cheek fine lines, forehead wrinkles, crow's feet wrinkles, and nasolabial folds, glabellar lines, under‐eye wrinkles, tear trough, and wrinkles at lip corners).

To assess structural changes in the dermis, ultrasound‐derived dermal density was measured using the DermaLab Combo system (Cortex Technology, Denmark) equipped with a 20 MHz high‐frequency ultrasound probe [12, 13, 14, 15]. Dermal density was quantified from ultrasound signal intensity within the selected dermal region using the proprietary image analysis algorithm of the SkinLab software. Higher values indicate greater dermal echogenicity and density of ultrasound‐reflecting dermal structures. Measurements were performed in the cheek region at a predefined anatomical location corresponding to the intersection of the vertical mid‐pupillary line and the horizontal line extending from the inferior margin of the nose. The measurement side (left or right cheek) was assigned according to a predefined randomization schedule and remained unchanged throughout the study. All ultrasound measurements were conducted by the same trained operator throughout the study to minimize inter‐operator variability and improve measurement consistency. At each time point, cheek dermal density measurement was obtained from each participant, and the group mean was calculated from all individual participant measurements. The operator was blinded to participant group allocation during image acquisition and analysis.

#### 2.5.5 Inflammatory Cytokines, Sirtuin Gene Expression, and NAD Metabolite Measurements

Blood samples were collected from 30 participants who were randomly selected from Clinical Study 1 at baseline and 12 weeks. Five cytokines (IFN‐γ, IL‐6, IL‐2, TNF‐α, hsCRP) were measured using flow cytometry (Biosciences, FACSCanto Flow Cytometer). They were quantified using a commercial multiplex bead‐based assay (Cytokine Combined Detection Kit, flow fluorescence luminescence method; Changsha Micro‐Meter Biotechnology, China).

In addition, the mRNA expression levels of SIRT1, SIRT3, and SIRT6 were quantified by quantitative real‐time reverse‐transcription PCR. Total RNA was extracted from whole blood with TRIzol reagent according to the manufacturer's instructions. RNA concentration and purity were assessed using a NanoDrop One spectrophotometer, and samples were stored at −80°C until analysis. Genomic DNA was removed using gDNA Eraser Mix Buffer, and reverse transcription was performed using 1 μg total RNA in a 20 μL reaction volume. Quantitative real‐time PCR was carried out with Universal SYBR Green qPCR Master Mix in a final volume of 20 μL containing 0.5 μL each of forward and reverse primers and 1 μL cDNA template. The amplification conditions were 95°C for 30 s, followed by 40 cycles of 95°C for 10 s and 60°C for 30 s. Melting curve analysis was performed using the instrument's default settings.

For measuring the level of NAD^+^ and total NAD (NAD^+^ and NADH), 20 μL of whole blood was incubated with the working solution, vortexed (2500 rpm, 10 min, 25°C), and centrifuged (13780 g, 10 min, 4°C). A 100‐μL aliquot of the supernatant was then dried under nitrogen, reconstituted in water, shaken (1500 rpm, 10 min, 25°C), and re‐centrifuged (2200 g, 10 min, 4°C). The resulting supernatant was subsequently analyzed via UHPLC–MS/MS.

#### 2.5.6 DNA Methylation‐Based Biological Age

A DNA methylation‐based physiological age prediction model was developed based on the Horvath epigenetic clock framework using publicly available whole‐blood DNA methylation datasets from healthy individuals spanning a broad age range [16, 17, 18, 19]. We curated multiple datasets containing whole blood samples from individuals across a broad age spectrum, ensuring participants were free from major diseases. Raw DNA methylation data (beta‐values) from Illumina Infinium HumanMethylation450K were downloaded. Standard preprocessing pipelines were applied, including background correction, probe filtering, and normalization. To identify CpG sites strongly correlated with chronological age, we calculated the Pearson correlation coefficient between each CpG's methylation beta‐value and the donor's age. Sites with the highest absolute correlation coefficients (|*r*| > 0.95) and statistically significant *p*‐values after multiple testing correction (FDR < 0.001) were selected as candidate age‐associated markers. Commonly identified loci included sites within the ELOVL2, FHL2, and KLF14 genes. From the initial candidate pool, we employed elastic net regression, a penalized linear model combining L1 (Lasso) and L2 (Ridge) regularization, to predict chronological age. The elastic net performs automatic feature selection (34 CpGs) while handling multicollinearity. Model training was conducted using packages like glmnet in R, with hyperparameters optimized via 10‐fold cross‐validation on the training set. The combined dataset was randomly split into a training set (50%) and a held‐out independent test set. Model performance was rigorously evaluated on the test set using standard metrics: the Pearson correlation coefficient (R) between predicted and chronological age, along with Median Absolute Deviation (MAD). The model achieves high explanatory power (*R* = 0.97) and low prediction error (MAD = 3.6 years) in independent validation.

### 2.6 Statistical Analysis

Statistical analyses were performed using SPSS version 21.0 (IBM) and GraphPad Prism version 9.0 (GraphPad Software). Data are presented as mean ± SD unless otherwise indicated. Normality and homogeneity of variance were assessed using the Shapiro–Wilk and Levene's tests, respectively. In vitro data from at least three independent experiments were analyzed using one‐way ANOVA with Tukey's multiple‐comparison test or, where appropriate, the Kruskal–Wallis test with Dunn's post hoc test. For image‐based assays, field‐level measurements were averaged within each biological replicate before analysis. Biomarkers measured at two time points were compared using paired *t*‐tests or Wilcoxon signed‐rank tests.

Clinical efficacy analyses were conducted as per‐protocol analyses. Participants who withdrew or were lost to follow‐up were excluded from endpoint‐specific analyses. Baseline between‐group comparisons were performed using independent‐samples *t*‐tests or Mann–Whitney *U*‐tests, as appropriate. All tests were two‐sided, and *p* < 0.05 was considered statistically significant.

## 3 Results

### 3.1 Determination of Polyphenols in FHLS

LC‐HRMS analysis identified a total of 78 compounds in FHLS, predominantly comprising flavonoids, phenolic acids, polyphenols, and triterpenoid saponins. Among these, 28 structurally distinct compounds were rigorously annotated as polyphenolic constituents based on established structural criteria [20, 21, 22]. The polyphenol compound identified in FHLS was summarized in Table S1.

### 3.2 In Vitro Anti‐Senescence Effects of FHLS in HSFs

**FIGURE 2:**
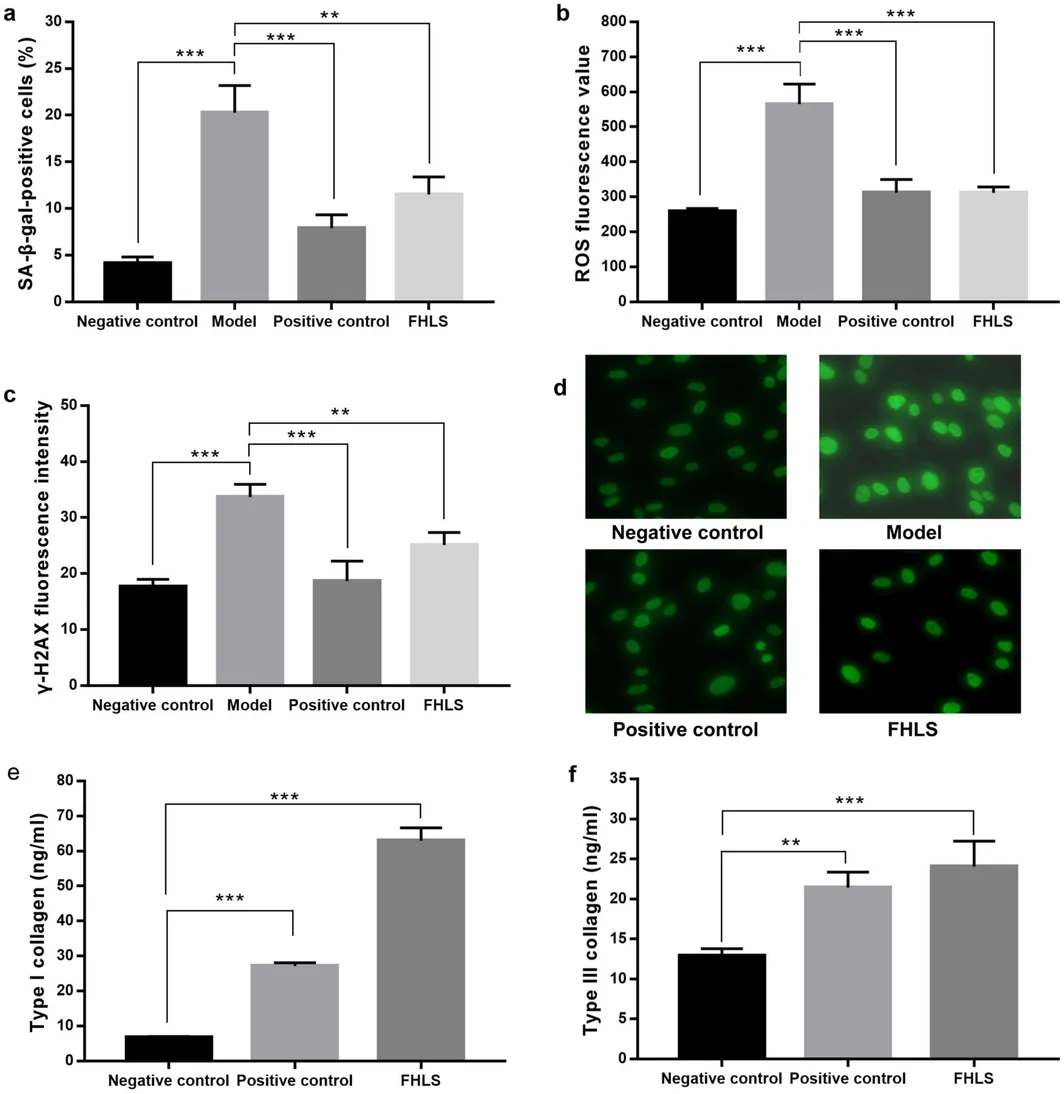
Molecular evidence of the anti‐senescence effects of FHLS in HSF. (a) SA‐β‐gal‐positive cells (%). (b) ROS fluorescence value. (c) γ‐H2AX fluorescence intensity. (d) Representative γ‐H2AX fluorescence images (green). Brighter green signal reflects higher DNA damage. (e, f) Type I and Type III collagen concentration (ng/ml). Asterisks indicate significant differences compared to the model group (for SA‐β‐gal activity, ROS, or γ‐H2AX foci) or the negative control (for Type I and Type III collagen): **p < 0.01, ***p < 0.001.

FHLS treatment at 2 mg/mL significantly reduced the proportion of SA‐β‐gal‐positive senescent cells (*p* < 0.01) (Figure 2a). Consistently, intracellular ROS levels were significantly attenuated following FHLS treatment relative to the model group (*p* < 0.001), to a level comparable to the positive control (Figure 2b). Immunofluorescence staining demonstrated that FHLS treatment substantially reduced the number of γ‐H2AX foci per nucleus, indicating a reduction in DNA damage (*p* < 0.001) (Figure 2c). Representative images of DNA damage under different treatment conditions are presented in Figure 2d. Moreover, ELISA analysis revealed that treatment with FHLS significantly upregulated the protein expression of both Type I and Type III collagen in HSFs compared to the negative control (*p* < 0.01), with a more pronounced effect relative to the positive control (Figure 2e,f).

### 3.3 Clinical Study 1 (FHLS‐Only)

#### 3.3.1 DNA Methylation‐Based Biological Age

**FIGURE 3:**
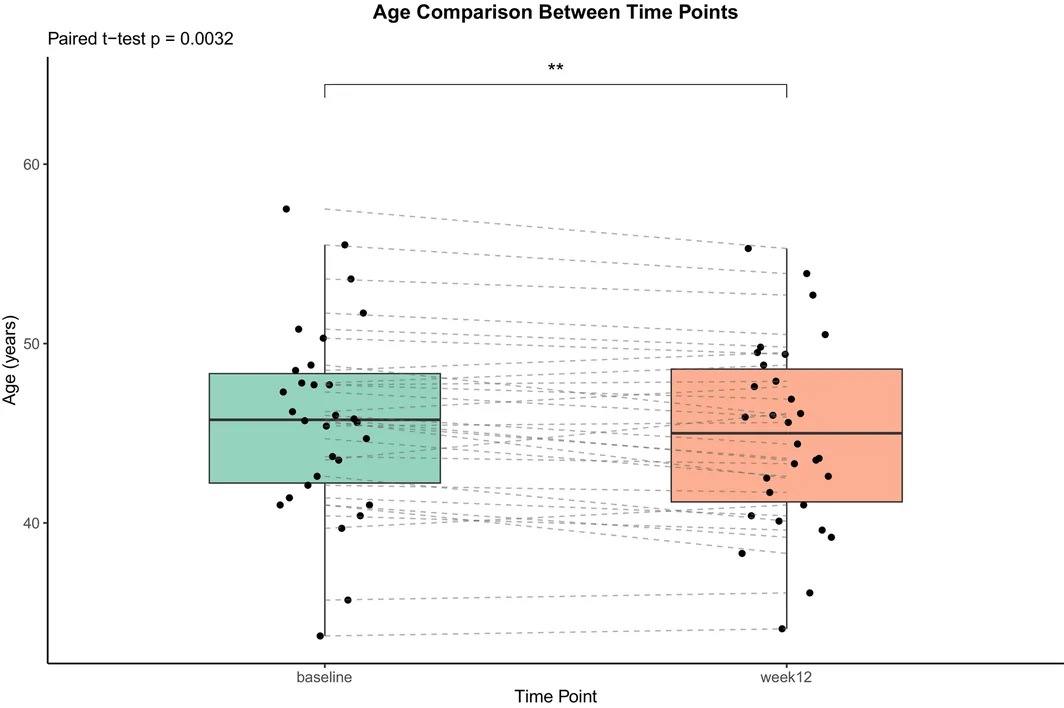
Epigenetic age reduction after 12 weeks of intervention. Asterisks denote significant differences from baseline: **p < 0.01.

The results indicated that 70% of the participants exhibited a reduction in DNA methylation‐based biological age following the intervention. From baseline to 12 weeks, the mean biological age decreased by an average of 0.84 years, which was statistically significant (*p* < 0.01). Notably, the reduction was observed consistently across the majority of subjects, suggesting a broadly applicable effect rather than being driven by a small subset of responders. In addition to the overall mean decrease, individual variability was observed, with the reduction up to 3 years of biological age. The observed reduction suggests a potential association with biological aging‐related biomarkers; however, the clinical significance of this finding requires further exploration (Figure 3).

#### 3.3.2 Skin Physiological Parameters

Following the 12‐week intervention, the dietary supplement significantly increased skin hydration while decreasing TEWL over the study period (all *p* < 0.001 vs. baseline), indicating enhanced moisture retention and an improved skin barrier function. Furthermore, skin radiance showed a significant and time‐dependent improvement, increasing from a baseline of 54.50 ± 3.98 SGU to 60.16 ± 3.90 SGU in 2 weeks (+10.38%), and further to 63.87 ± 3.95 in 12 weeks (+17.19%). A significant increase in cheek skin elasticity (R2) was observed at 2, 4, and 12 weeks (all *p* < 0.001 vs. baselines). Concurrently, skin firmness (F4) demonstrated significant decreases at all follow‐up assessments, indicating improved skin firmness (Table 1).

**TABLE 1:** Changes in skin physiological parameters at different time points (*N* = 87).

Variable	Baseline	2 weeks	4 weeks	12 weeks
Skin hydration (C.U.)	31.00 ± 8.26	45.99 ± 8.69 (+48.36%)***	48.81 ± 8.27 (+57.43%)***	50.36 ± 8.29 (+62.46%)***
TEWL (g/m^2^/h)	14.13 ± 2.49	12.49 ± 2.29 (−11.55%)***	11.99 ± 1.97 (−15.09%)***	11.49 ± 1.72 (−18.69%)***
Skin radiance (SGU)	54.50 ± 3.98	60.16 ± 3.90 (+10.38%)***	62.75 ± 4.09 (+15.14%)***	63.87 ± 3.95 (+17.19%)***
Skin elasticity (R2)	0.61 ± 0.06	0.65 ± 0.06 (+7.08%)***	0.69 ± 0.06 (+14.20%)***	0.699 ± 0.07 (+15.16%)***
Skin firmness (F4)	16.21 ± 2.35	14.41 ± 2.21 (−11.07%)***	14.03 ± 2.24 (−13.47%)***	13.80 ± 1.95 (−14.85%)***

*Note:* Data is presented as mean ± SD. Values in parentheses indicate the mean percentage change from baseline. *** denotes a significant difference from baseline at *p* < 0.001.

**FIGURE 4:**
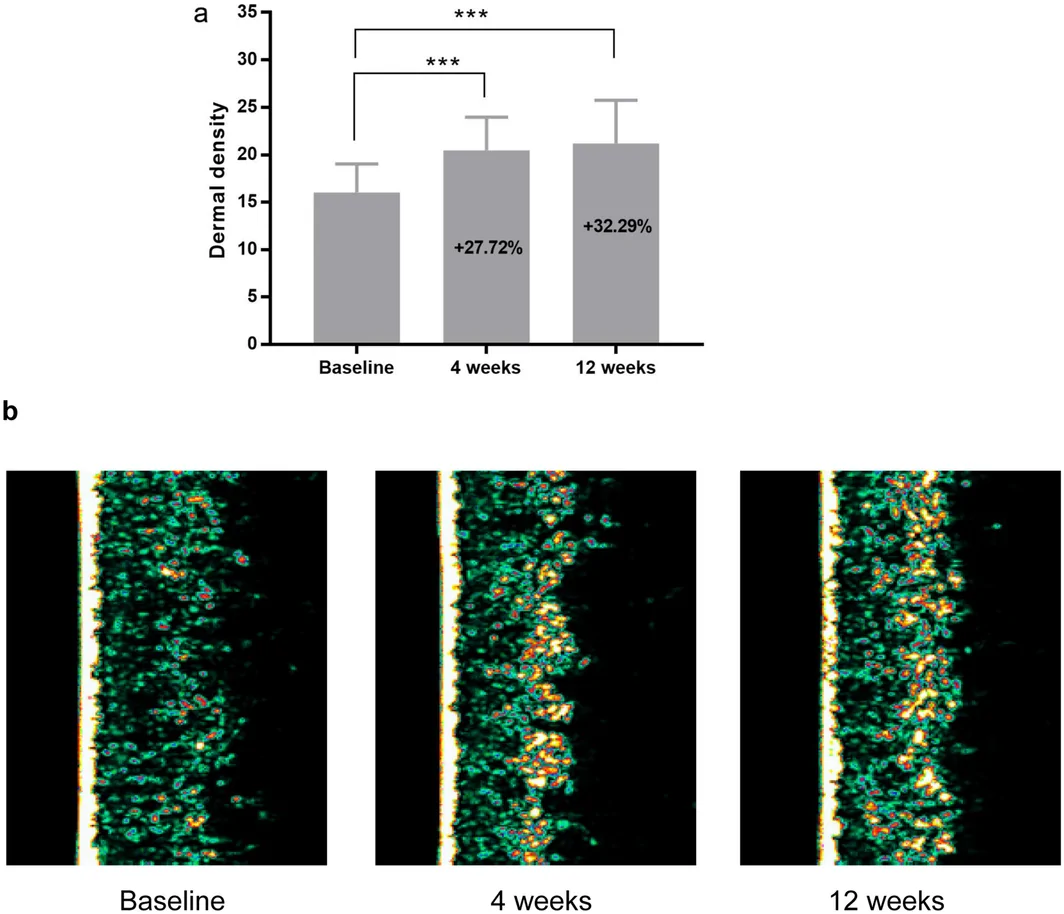
Increase in dermis density in the cheek over time. (a) Quantification of dermal density after intervention. *** denotes a significant difference from baseline at *p* < 0.001. (b) Representative ultrasound images at baseline, 4, and 12 weeks. Colors represent ultrasound signal intensity, which reflects dermal density and is positively associated with dermal collagen content, with darker green indicating lower dermal density and collagen content while brighter yellow indicating higher dermal density and collagen content.

Dermal density was assessed using signal intensity, an ultrasound‐derived parameter reflecting dermal echogenicity and structural characteristics that may be associated with collagen‐related skin properties, with higher signal intensity indicating greater collagen abundance in the dermis [23, 24]. Compared with baseline, dermal density in the cheek increased significantly, rising by 27.72% at 4 weeks (*p* < 0.001) and by 32.29% at 12 weeks (*p* < 0.001), demonstrating progressive improvement over time (Figure 4a). Representative ultrasound images of the cheek from a 44‐year‐old female participant are shown in Figure 4b.

**FIGURE 5:**
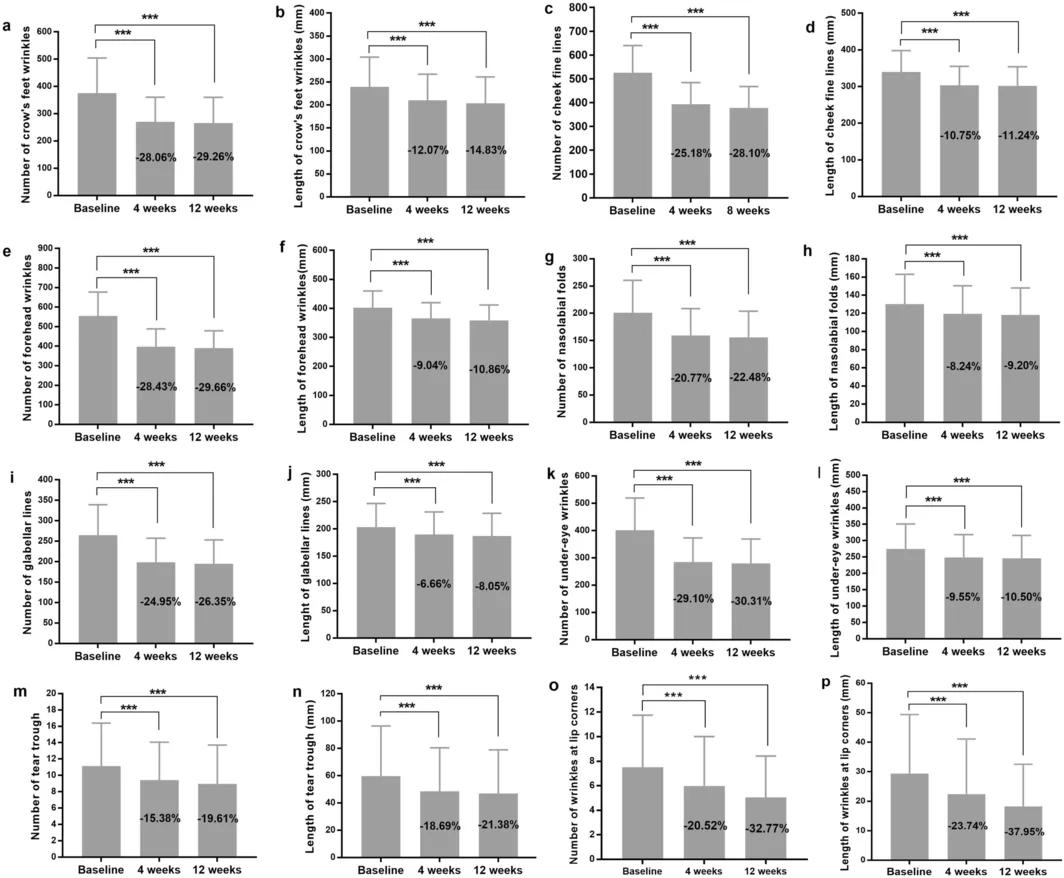
Facial wrinkle improvement after FHLS intervention. (a–p) Wrinkle indicators (number and length) measured at baseline, 4, and 12 weeks. (a, b) crow's feet wrinkles; (c, d) Cheek fine lines; (e, f) forehead wrinkles; (g, h) nasolabial folds; (i, j) glabellar lines; (k, l) under‐eye wrinkles; (m, n) tear trough lines; and (o, p) wrinkles at lip corners, respectively *** denotes a significant difference from baseline at *p* < 0.001. Changes from baseline are shown as percentages within the gray bars. Negative values indicate a reduction from baseline, corresponding to improvement.

**FIGURE 6:**
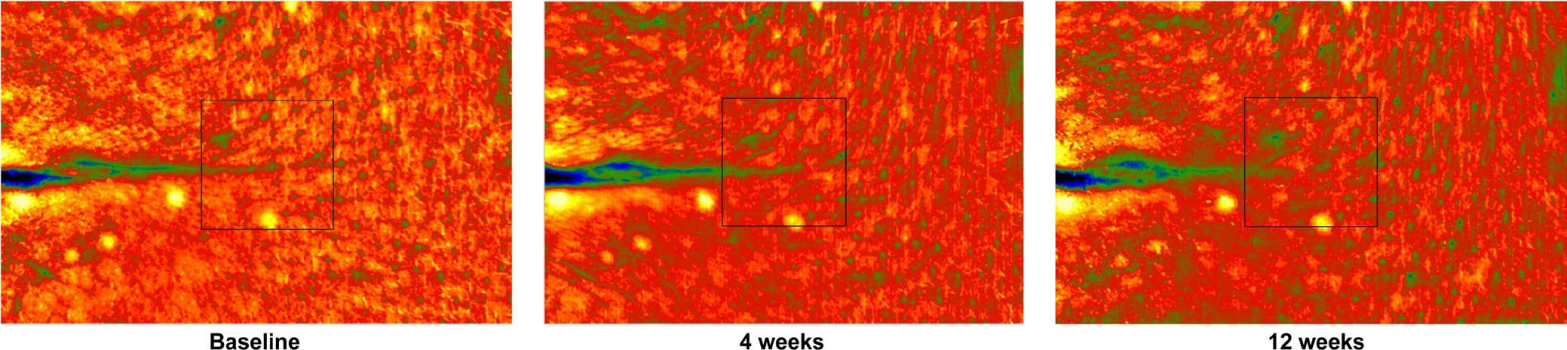
PRIMOS CR 3D images depicting changes in crow's feet wrinkles in a 47‐year‐old female participant. Different colors represent the skin surface height: bright yellow‐to‐red indicates raised areas and dark green‐to‐blue indicates depressed areas.

Significant reductions in both the number and length of all eight assessed facial wrinkle types, including crow's feet wrinkles, cheek fine lines, forehead wrinkles, nasolabial folds, glabellar lines, under‐eye wrinkles, tear trough, and wrinkles at lip corners, were consistently observed at 4 and 12 weeks compared to baseline (all *p* < 0.001). In particular, the wrinkle number was reduced by 19.61%–32.77%, and the wrinkle length decreased by 8.05%–37.96% (Figure 5). Primos CR 3D image of crow's feet wrinkles changes following consumption at 4 and 12 weeks was presented in Figure 6.

#### 3.3.3 Inflammatory Cytokines, Sirtuin Genes and NAD Metabolites Measurements

**FIGURE 7:**
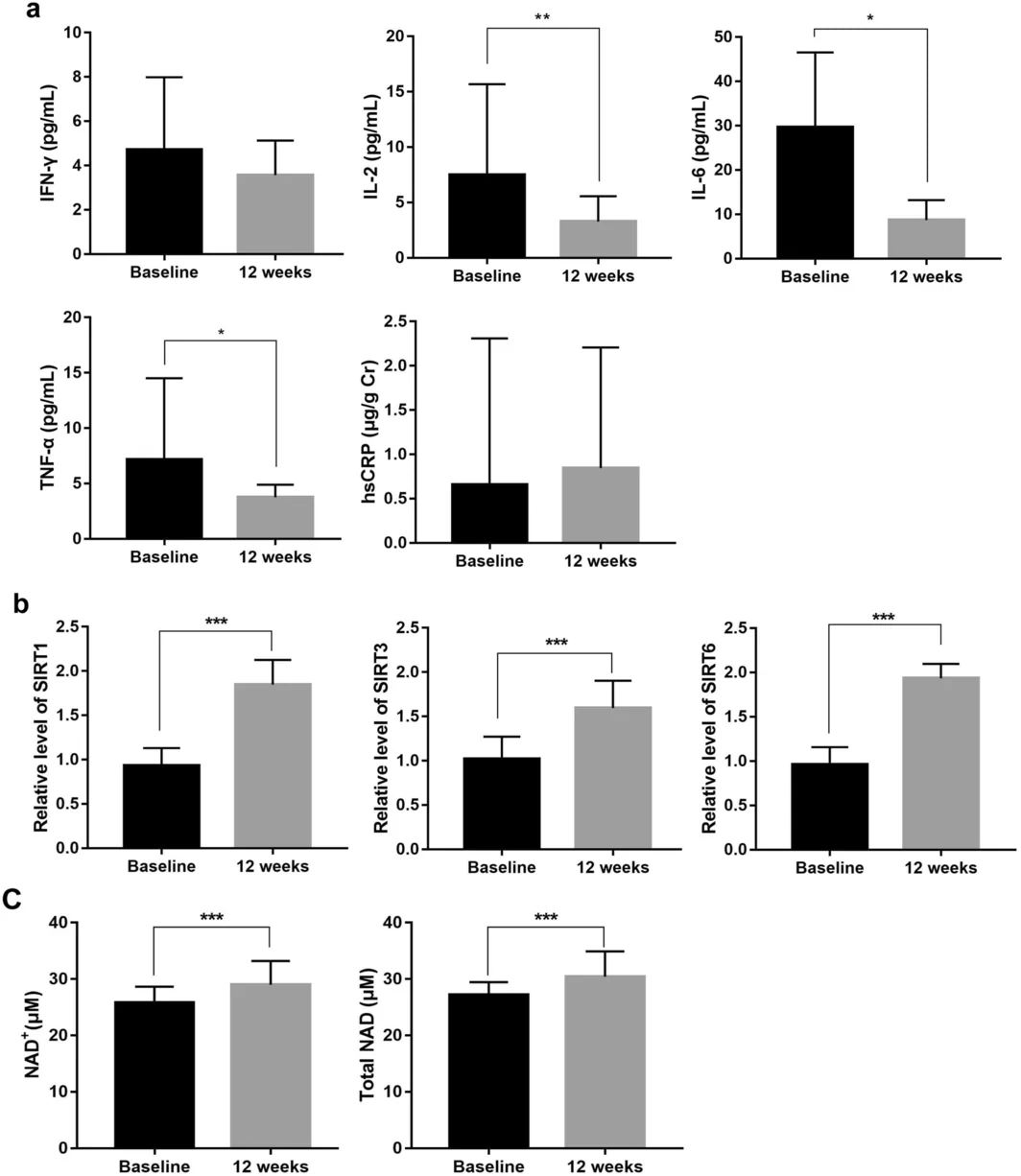
Changes in inflammatory cytokines, sirtuin gene expression, NAD metabolites in blood samples following a 12‐week intervention. Data are presented as median with interquartile range. (a) inflammatory cytokine levels (IFN‐γ, IL‐2, IL‐6, TNF‐α, and hsCRP); (b) relative levels of SIRT1, SIRT3, and SIRT6; (c) NAD^+^ and total NAD levels. Asterisks denote significant differences from baseline: *p < 0.05, **p < 0.01, and ***p < 0.001.

Levels of inflammatory cytokines, expression of sirtuin genes (SIRT1, SIRT3, and SIRT6), NAD^+^, and total NAD were assessed at baseline and after 12 weeks of FHLS supplementation. Overall, a significant downregulation of several inflammatory cytokines was observed at 12 weeks. Specifically, the concentrations of IL‐6, IL‐2, and TNF‐α were significantly reduced compared with baseline (*p* < 0.05 for IL‐6 and TNF‐α; *p* < 0.01 for IL‐2). The decrease was most pronounced for IL‐2, which showed a reduction of 55.37% (*p* < 0.01). Although levels of IFN‐γ and hsCRP also exhibited a declining trend in 12 weeks, the changes did not reach statistical significance. The expression of sirtuin genes SIRT1, SIRT3, and SIRT6 significantly increased compared to baseline (*p* < 0.001), with SIRT1 rising by 70.46%, SIRT3 by 53.28%, and SIRT6 by 85.32%. In parallel, both NAD^+^ and total NAD levels showed significant increases after 12 weeks of intervention, with NAD^+^ increasing by 14.16% and total NAD increasing by 15.47% compared with baseline (both *p* < 0.001), indicating enhanced cellular NAD^+^ metabolism following FHLS supplementation (Figure 7).

### 3.4 Clinical Study 2 (Combination Regimen)

#### 3.4.1 Topical Skincare Plus Oral FHLS Versus Topical Skincare Group

A total of 70 healthy female participants were enrolled in this study. The average age of skincare and skincare + FHLS groups was 48.30 ± 2.77 and 47.21 ± 3.08 years. Four participants (two per group) were excluded due to non‐responsiveness, resulting in 66 cases for final analysis.

Both interventions produced significant improvements in crow's feet wrinkles over 12 weeks; however, the combination regimen demonstrated a consistently greater magnitude of benefit than skincare alone across all post‐baseline assessments (Table 2). In terms of wrinkle number, the combined group achieved reductions of 32.58%, 34.44%, and 37.84% at Weeks 2, 4, and 12, respectively, compared with 24.43%, 26.13%, and 27.17% in the skincare group. A similar pattern was observed for wrinkle length, where the combination regimen showed decreases of 16.33%, 18.01%, and 20.65%, whereas the skincare group showed smaller reductions of 10.67%, 12.14%, and 14.42% at the corresponding time points. Notably, the superiority of the combined intervention was evident as early as Week 2 and was sustained through Week 12, suggesting not only a faster onset of action but also a greater cumulative anti‐wrinkle effect. In addition, both groups exhibited progressive within‐group improvement from Week 2 to Week 12, indicating that continued use enhanced efficacy over time. Collectively, these findings show that while skincare alone significantly improved crow's feet wrinkles, the addition of FHLS resulted in significantly greater and more durable reductions in both wrinkle number and length.

**TABLE 2:** Percent changes in crow's feet wrinkle number and length from baseline over the 12‐week intervention period.

Time point	Crow's feet wrinkles number		Crow's feet wrinkles length	
	Skincare group (*N* = 33)	Skincare + FHLS group (*N* = 33)	Skincare group (*N* = 33)	Skincare + FHLS group (*N* = 33)
Week 2	−24.43%	−32.58%	−10.67%	−16.33%
Week 4	−26.13%	−34.44%	−12.14%	−18.01%
Week 12	−27.17%	−37.84%	−14.42%	−20.65%

*Note:* Data are presented as percentage changes from baseline. Negative values indicate a reduction in wrinkle number or length.

**FIGURE 8:**
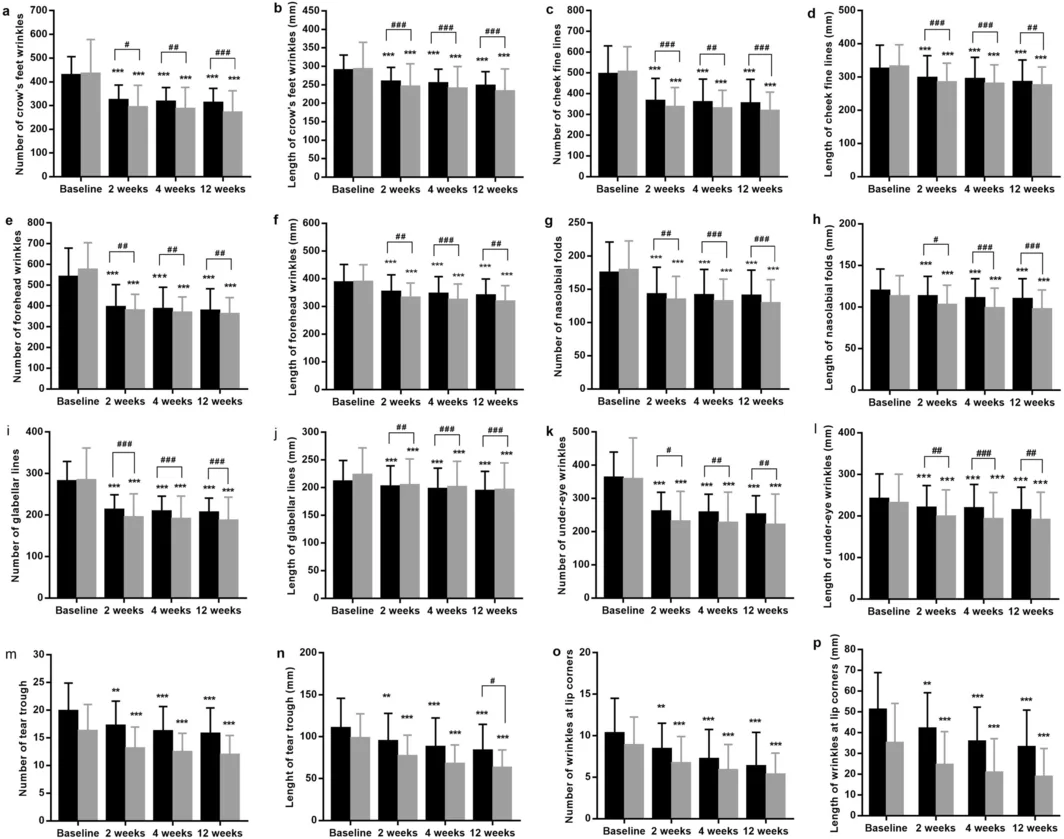
Facial wrinkle improvement. (a–p) Changes in wrinkle number and length at baseline, 2, 4, and 12 weeks in the Skincare group (black bars) and Skincare + FHLS group (gray bars). (a, b) crow's feet wrinkles; (c, d) cheek fine lines; (e, f) forehead wrinkles; (g, h) nasolabial folds; (i, j) glabellar lines; (k, l) under‐eye wrinkles; (m, n) tear trough lines; and (o, p) wrinkles at lip corners, respectively. * indicates a significant difference versus baseline (*p* < 0.05, **p < 0.01, ***p < 0.001), whereas # indicates a significant difference between groups (*p* < 0.05, *p* < 0.01, *p* < 0.001).

Other skin assessments, including wrinkle number and length for cheek fine lines, forehead wrinkles, and nasolabial folds, also showed significant improvement over the 12‐week study (*p* < 0.01, Figure 8). The skincare + FHLS regimen demonstrated statistically superior efficacies for most assessed facial wrinkle parameters, including cheek fine lines, forehead wrinkles, nasolabial folds, glabellar lines, under‐eye wrinkles, tear trough lines, except for wrinkles at the lip corners. This indicates that augmenting the dietary supplement with the topical skincare regimen provides a significantly enhanced anti‐wrinkle benefit.

Skin hydration, radiance, and elasticity (R2) increased progressively over time in both groups, with significantly larger improvements observed in the skincare + FHLS group. Conversely, TEWL and skin firmness (F4), where a lower value indicates improvement, showed a time‐dependent reduction, with the skincare + FHLS group achieving a significantly greater decrease (Table 3). The results collectively demonstrate that the intervention improved skin barrier function, hydration, and biomechanical properties.

**TABLE 3:** Changes in skin physiological parameters.

Variable	Skincare group(*N* = 33)		Skincare + FHLS group (*N* = 33)		*p* (between groups)
	Mean ± SD	Change from baseline (%)	Mean ± SD	Change from baseline (%)	
Skin hydration (C.U.)					
Baseline	25.91 ± 5.09	—	25.20 ± 5.36	—	—
2 weeks	39.38 ± 4.55***	+51.95%	41.92 ± 5.71***	+66.34%	0.006
4 weeks	41.16 ± 4.36***	+58.85%	46.05 ± 5.33***	+82.76%	< 0.001
12 weeks	46.18 ± 5.77***	+78.20%	49.59 ± 5.62***	+96.81%	0.006
TEWL (g/m^2^/h)					
Baseline	15.88 ± 3.12	—	16.25 ± 1.85	—	—
2 weeks	14.19 ± 2.77***	−10.64%	13.73 ± 1.46***	−15.50%	0.001
4 weeks	13.54 ± 2.70***	−14.70%	13.11 ± 1.44***	−19.32%	0.012
12 weeks	12.91 ± 1.99***	−18.67%	12.13 ± 1.48***	−25.37%	0.003
Skin radiance (SGU)					
Baseline	56.13 ± 3.74	—	56.41 ± 3.26	—	—
2 weeks	62.86 ± 3.61***	+11.98%	65.88 ± 3.28***	+16.78%	< 0.001
4 weeks	63.19 ± 3.28***	+12.58%	67.97 ± 3.25***	+20.48%	< 0.001
12 weeks	64.57 ± 3.30***	+15.03%	69.69 ± 3.60***	+23.53%	< 0.001
Skin elasticity (R2)					
Baseline	0.661 ± 0.065	—	0.647 ± 0.047	—	—
2 weeks	0.692 ± 0.054***	+4.77%	0.716 ± 0.039***	+10.66%	< 0.001
4 weeks	0.702 ± 0.050***	+6.25%	0.747 ± 0.031***	+15.45%	< 0.001
12 weeks	0.703 ± 0.049***	+6.32%	0.767 ± 0.031***	+18.39%	< 0.001
Skin firmness (F4)					
Baseline	16.34 ± 2.22	—	15.95 ± 1.76	—	—
2 weeks	15.09 ± 1.96***	−7.65%	13.46 ± 1.48***	−15.60%	< 0.001
4 weeks	14.78 ± 1.76***	−9.55%	12.62 ± 1.28***	−20.90%	< 0.001
12 weeks	14.21 ± 1.92***	−13.02%	12.08 ± 1.48***	−24.27%	< 0.001

*Note:* Data are presented as mean ± SD. *** denotes a significant difference from baseline at *p* < 0.001.

**FIGURE 9:**
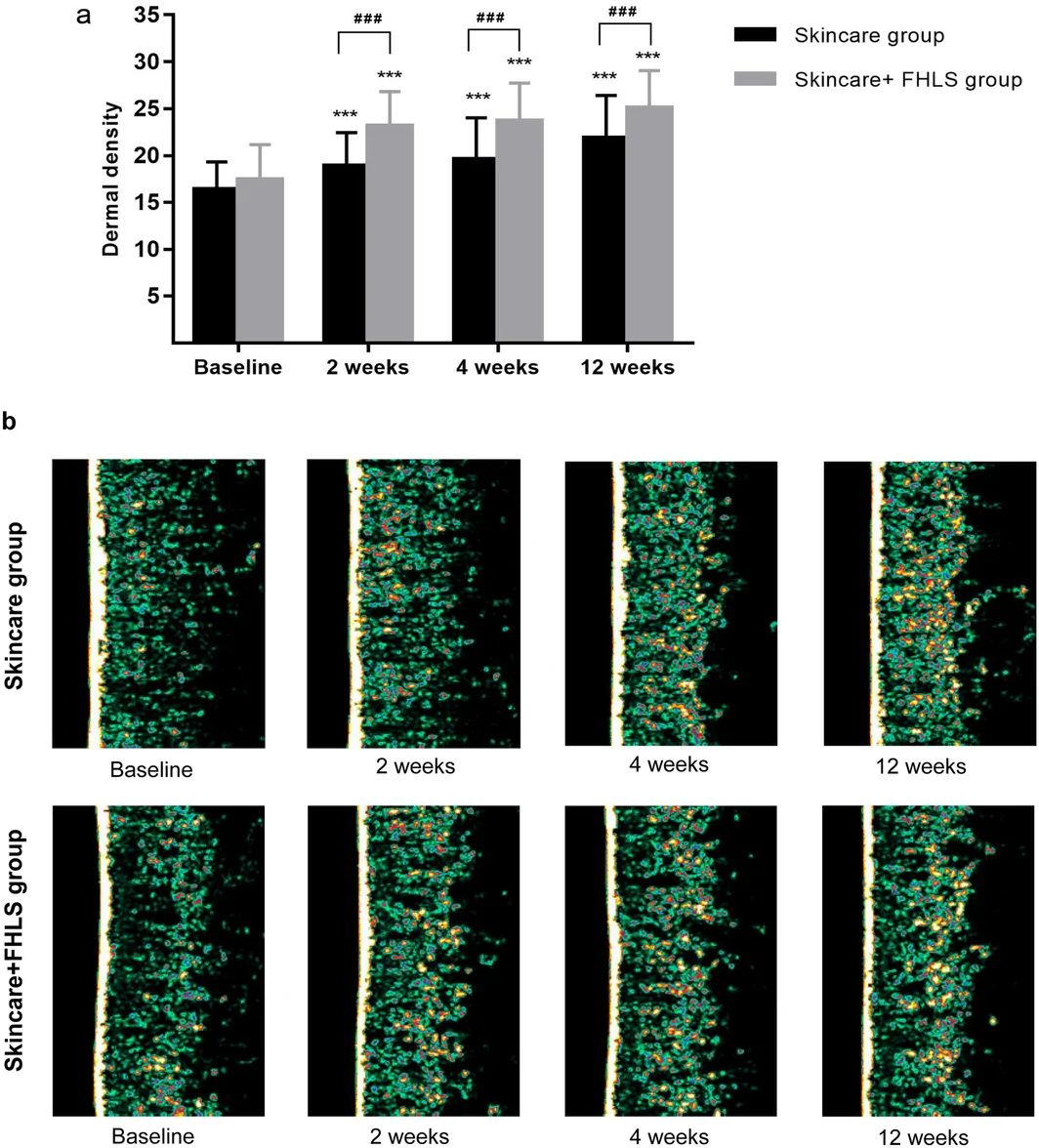
Dermal density changes following skincare and skincare + FHLS intervention (a) Quantification of dermal density after intervention, Symbols denote significance: ***p < 0.001 vs. baseline; ^###^ *p* < 0.001 between groups. (b) Representative ultrasound images. Colors represent ultrasound signal intensity, which reflects dermal density and is positively associated with dermal collagen content, with darker green indicating lower dermal density and collagen content while brighter yellow indicating higher dermal density and collagen content.

Dermal density in the cheek showed a significant, time‐dependent increase from baseline in both groups at all follow‐ups (all *p* < 0.001 vs. baseline). The mean density improved from 16.35 to 21.89 (a 33.89% increase) in the skincare group and from 17.44 to 25.11 (a 43.95% increase) in the skincare + FHLS group. Notably, the improvement was consistently and significantly greater in the skincare + FHLS group at each post‐baseline assessment (all *p* < 0.001 for between‐group comparisons) (Figure 9).

## 4 Discussion

The present study provides evidence that FHLS is associated with changes across multiple skin‐related endpoints and selected biomarkers relevant to aging biology. Skin aging is not merely a cosmetic or organ‐confined phenomenon; rather, it reflects systemic, interrelated biological processes, including cellular senescence, oxidative stress, extracellular matrix remodeling/deterioration, and chronic low‐grade inflammation [25, 26]. Consequently, no single endpoint can fully capture the multidimensional nature of change, and interpretation is strengthened by combined evidence across complementary clinical phenotypes and mechanistic biomarkers. A more realistic view is that several pathways are involved at once, often influencing each other rather than acting independently. In this study, FHLS, an herbal formulation adapted from the classical prescription *Qiongyu* Paste and supplemented with PQQ, astaxanthin, and apple polyphenols, showed coordinated effects across different types of evidence including in vitro mechanistic assays, objective clinical skin assessments, and selected systemic biomarkers. Improvements in skin hydration and barrier function can be directly tied to better moisture retention. However, structural changes like greater dermal density and wrinkle reduction involve a more complex interplay of mechanisms. These outcomes do not stem from a single cause, but rather from the convergence of multiple molecular pathways‐including reduced cellular senescence, decreased oxidative stress, and enhanced synthesis of extracellular matrix, as demonstrated in mechanistic assays. These findings collectively reveal an overall pattern, where changes at the cellular level, the tissue level, and the systemic level tend to move in the same direction. The value of the present work therefore lies not only in the observed improvement in skin‐related outcomes, but also in the integration of mechanistic, phenotypic, and exploratory systemic evidence within a unified evaluation framework.

Aging involves the gradual buildup of senescent cells and the disruption of tissue homeostasis, and these changes are particularly evident in the skin, where both intrinsic processes and external exposures contribute to functional and structural decline [27, 28]. Senescent cells accumulate over time as part of a stress response, often linked to DNA damage accumulation, metabolic alterations, and increased production of ROS [29, 30, 31] In parallel, NAD+‐dependent sirtuin pathways, particularly SIRT1, SIRT3 and SIRT 6, have been increasingly recognized as key regulators of cellular stress resistance, mitochondrial homeostasis, genomic stability, and cellular senescence. In the context of skin aging, these pathways intersect with oxidative stress responses, DNA damage repair, and dermal fibroblast function, thereby contributing to extracellular matrix maintenance and structural integrity [32]. The skin is especially affected because it is continuously exposed to environmental stressors in addition to internal aging processes. Among these, ultraviolet radiation stands out, as it can induce both DNA damage and oxidative stress, which in turn promotes cellular senescence [33]. This is also associated with decreased collagen production [34]. In this context, the reduction in SA‐β‐gal‐positive senescent cells, intracellular ROS levels, and γ‐H2AX foci observed after FHLS treatment can be coherently interpreted, as follow patterns already described in skin aging biology [35]. The observed upregulation of sirtuin gene expression in the present study is directionally consistent with this mechanistic framework, although the current data does not establish a direct causal role for sirtuin signaling in mediating the observed effects [36, 37, 38]. Central to these structural improvements is the increased synthesis of Type I and III collagen. Its significance is paramount, as it targets a trial of well‐established pathophysiological features of skin aging: dermal atrophy, impaired elastic fiber function, and progressive wrinkle development [39]. Collectively, these findings suggest that FHLS may act on several interconnected cellular processes involved in skin aging, which helps explain the nature of the clinical observations of the clinical findings, even if the exact contribution of each pathway is not fully separated. The clinical findings from the FHLS‐only trial were generally in line with the mechanistic observations and pointed to improvements at both the functional and structural levels of the skin over the 12‐week intervention. The increases in skin hydration together with the reduction in TEWL indicate an improvement in skin condition and barrier function, which are often impaired in aged skin [40, 41, 42]. More importantly, the changes in skin radiance, elasticity (R2), firmness‐related parameters, dermal density, and wrinkle measures suggest that the effects of FHLS were not confined to surface‐level moisturization. These outcomes appear to extend to features that are more closely related to dermal integrity. Skin aging is known to involve progressive degradation of the dermal extracellular matrix, including the loss and disorganization of collagen and elastin fibers, so improvements in elasticity, dermal density, and wrinkle outcomes are biologically more informative than changes in surface hydration alone [43, 44, 45, 46, 47, 48, 49]. In this setting, the parallel increase in dermal density observed clinically and the enhancement of Type I and III collagen secretion seen in vitro seem to move in the same direction, which supports the plausibility of an ECM‐related effect. It is also worth noting that previous studies of individual FHLS‐related ingredients, when used alone, have shown limited or inconsistent effects on hydration or TEWL [36, 37, 38]. Collectively, it is a single‐ingredient explanation, favoring the conclusion that the effects stem from the combined‐and potentially additive‐actions of the FHLS blend.

Beyond the cutaneous findings, this study also provides preliminary evidence that FHLS may influence selected systemic processes relevant to aging biology. While these observations are directionally consistent with mechanisms implicated in aging biology, they should be regarded as exploratory because the biomarker analyses were performed without a placebo‐controlled comparator. Notably, FHLS supplementation was associated with a statistically significant reduction in DNA methylation‐based biological age. The observed reduction in DNA methylation‐based biological age should be interpreted in the context of previous human intervention studies [50, 51, 52]. The DO‐HEALTH trial, as an example of a nutritional intervention, reported significant improvements in blood‐based epigenetic aging measures corresponding to approximately 2.9–3.8 months of biological age change following omega‐3 supplementation, vitamin D administration, and exercise up to 3 years. In the TRIIM trial, pharmacological intervention for 12 months resulted in reductions of 2.16–3.73 years across multiple blood‐derived epigenetic clocks. Similarly, an 8‐week diet and lifestyle intervention was associated with a 1.96‐year reduction in biological age based on saliva‐derived DNA methylation analysis. Although direct comparisons across studies are limited by differences in study design, populations, biological samples, and epigenetic clocks, these findings indicate that DNA methylation‐based biological age is responsive to diverse interventions across clinical settings.

The reductions in IL‐6, IL‐2, and TNF‐a are notable in the context of immunosenescence and inflammaging, which are increasingly recognized as important contributors to progressive tissue dysfunction with aging [53, 54]. Likewise, the observed increase in blood NAD+ levels is directionally consistent with pathways involved in cellular repair, stress responses, and metabolic homeostasis [55]. Given the NAD^+^‐dependence of sirtuin signaling, the concurrent increase in SIRT1, SIRT3, and SIRT6 gene expression further supports the possibility that FHLS may modulate NAD^+^‐sirtuin‐related pathways involved in metabolic regulation and cellular resilience. Although these systemic findings should be interpreted cautiously, they extend the significance of the study beyond skin appearance alone and suggest that some of the clinical improvements may occur within a broader biological context. This broader perspective is further supported by the randomized parallel trial, in which the combination of FHLS oral supplementation with topical skincare produced greater improvements in clinical signs of skin aging than topical skincare alone, particularly for elasticity, dermal density, and wrinkle‐related outcomes. These findings are consistent with the combination strategy, where oral supplementation may influence systemic biological processes associated with skin aging, while topical skincare acts directly on cutaneous tissues. Although the specific mechanisms underlying these effects remain to be fully elucidated, these systemic biomarker findings together with the greater outcomes observed with the combined regimen support the translational relevance of this approach and warrant further investigation in larger placebo‐controlled studies.

In this study, several strengths should be noticed. A key strength is the use of a multi‐level evaluation approach, combining mechanistic experiments in HSFs with the data do not support the objective, instrument‐based clinical assessments, which helps support the biological relevance of the observed changes. The clinical design also adds value, as it includes both an open‐label supplementation study tracking time‐dependent changes in skin parameters and selected biomarkers, and a randomized, parallel‐controlled trial assessing the added benefit of oral supplementation alongside topical skincare. In addition, skin outcomes were measured using validated non‐invasive instruments under controlled conditions, which improves consistency, and the FHLS formulation itself was standardized with quality control measures such as HPLC, supporting reproducibility.

It is also important to acknowledge the limitations of this study for balanced consideration. The FHLS‐only study lacked placebo control, so expectation effects, seasonal variation, and regression to the mean cannot be excluded. Analyses were based on per‐protocol populations, which may introduce attrition bias, and participant blinding was not implemented in the randomized study, leaving room for performance bias. The systemic biomarker findings should also be interpreted cautiously, given the small sample size, limited time points, and absence of placebo control. In addition, the study population was limited to healthy Chinese middle‐aged women, which restricts generalizability, and the 12‐week duration does not address long‐term effects or safety. Furthermore, DNA methylation‐based biological age changes observed in the present study are in line with those reported in previous human intervention studies; however, interpretation is limited by the absence of an established threshold for clinically meaningful change and the inherent variability of epigenetic clock‐based measurements. Finally, the in vitro models and dosing conditions may not fully reflect in vivo complexity, so further mechanistic work is needed to better understand how the individual components contribute to the overall effects.

## 5 Conclusions

In summary, FHLS, a dietary herbal mixture, was associated with coordinated changes across cellular assays, objective skin measurements, and selected systemic biomarkers. The randomized trial further suggests that adding oral FHLS to topical skincare can improve several skin outcomes compared with topical treatment alone. Taken together, these results support the feasibility and translational relevance of a combined oral–topical approach and warrant confirmation in larger, placebo‐controlled studies with blinded assessments.

## Author Contributions

Conceptualization: Jie‐Hua Chen, Jun Ma. Methodology: Jie‐Hua Chen, Jun Ma. Validation: Liugang Ding, Pingping Lv. Formal analysis: Jie‐Hua Chen, Meiling Tai, Ran Yin, Yi Yu, Yunru Huang. Investigation: Erman Wang, Huan Liu, Jiaojiao Fu. Resources: Liugang Ding, Pingping Lv, Meiling Tai, Wenzhi Li, Erman Wang. Data curation: Jinluan Zheng, Erman Wang, Huan Liu, JiaoJiao Fu. Writing‐original draft: Jie‐Hua Chen, Ran Yin, Erman Wang. Writing‐review and editing: Jie‐Hua Chen, Jun Ma. Visualization: Erman Wang, Huan Liu, Jiaojiao Fu. Supervision: Jie‐Hua Chen, Jun Ma. Project administration: Jie‐Hua Chen. Funding acquisition: Jun Ma.

## Funding

This research was supported by the National Key R&D Program of China (2024YFE0104900, 2024YFF1106000).

## Ethics Statement

The clinical study was conducted in accordance with the ethical principles of the Declaration of Helsinki. The study protocol and procedures were approved by the Ethics Committee of Guangdong Daily Chemical Industry (Approval No. GDIRB [2025]6‐0157A), and written informed consent was obtained from all participants participating in the study.

## Conflicts of Interest

Some authors are employees of Infinitus (China) Company Ltd., which provided the study product. The authors declare that they have no other competing interests.

## Supporting information


**Table S1:** Polyphenols identified in FHLS by LC‐HRMS analysis.

## Data Availability

The data that support the findings of this study are available from the corresponding author upon reasonable request.
