# Supplementary material for: Effects of a Dietary Supplement on Systemic Aging Related Biomarkers and Skin Aging Outcomes in Middle‐Aged Women: In Vitro and Clinical Studies
Source: J Cosmet Dermatol. 2026 Sep 6;25(9):e71175. doi: 10.1111/jocd.71175 (PMC13547764; doi:10.1111/jocd.71175)
Supplement: Supplementary file 1 [file JOCD-25-e71175-s001.docx]

**Supplementary Materials**

Determination of polyphenols in FuHuoLvShi (FHLS)

The compounds in FHLS were profiled by Liquid Chromatography-High Resolution Mass Spectrometry(LC- HRMS). For sample preparation, certain amount of FHLS was fully dissolved in methanol and extracted ultrasonically at room temperature for 30 min. Then, the extract was centrifuged at 4696 g for 10 min, and the resulting supernatant was passed through a 0.22 μm membrane filter before injection. Chromatographic separation was achieved on a C18 reversed-phase column (2.1 × 100 mm, 1.9 μm) with mobile phase consisting of 0.1% formic acid in water (solvent A) and 0.1% formic acid in acetonitrile (solvent B). Gradient elution was performed with solvent B maintained at 5% during 0–2 min, linearly increased from 5% to 95% over 2–42 min, held at 95% from 42–47 min, rapidly decreased from 95% to 5% over 47–47.1 min, and then equilibrated at 5% from 47.1 to 50 min. The flow rate was set at 0.3 mL/min, with the column temperature maintained at 40 °C and the injection volume fixed at 3 μL. Mass spectrometric acquisition was performed using an electrospray ionization source with a spray voltage of 3.5 kV, capillary temperature of 320 °C, sheath gas flow of 35 arb, and auxiliary gas flow of 10 arb. Data were collected in Full MS-ddMS² mode over an m/z range of 100–1500, with stepped normalized collision energies of 20, 40, and 60. The resulting LC-HRMS datasets were processed for putative identification of FHLS-derived chemical constituents. Overall, the analysis identified a total of 78 compounds in FHLS, predominantly including flavonoids, phenolic acids, polyphenols, and triterpenoid saponins. These compounds were then further classified into recognized polyphenol subclasses, resulting in a total of 28 structurally distinct compounds that were rigorously annotated as polyphenolic constituents. All results were summarized in Table S1.

| **Compound** | **Formula** | **Polyphenol Class** |
| --- | --- | --- |
| 4-Coumaric acid | C_9_H_8_O_3_ | Phenolic acid (hydroxycinnamic) |
| Ferulic acid | C_10_H_10_O_4_ | Phenolic acid (hydroxycinnamic) |
| Chlorogenic acid | C_16_H_18_O_9_ | Phenolic acid (caffeoylquinic acid) |
| Neochlorogenic acid | C_16_H_18_O_9_ | Phenolic acid (caffeoylquinic acid) |
| 2-Coumaroylquinic acid | C_16_H_18_O_8_ | Phenolic acid derivative |
| 4,5-Dicaffeoylquinic acid | C_25_H_24_O_12_ | Polyphenolic acid (dicaffeoyl) |
| Gallic acid | C_7_H_6_O_5_ | Phenolic acid (hydroxybenzoic) |
| Quercetin | C_15_H_10_O_7_ | Flavonol |
| Taxifolin | C_15_H_12_O_7_ | Flavanonol |
| Datiscetin | C_15_H_10_O_6_ | Flavonoid |
| Kaempferol 3-robinobioside | C_27_H_30_O_15_ | Flavonol glycoside |
| Isorhamnetin 3-robinobioside | C_28_H_32_O_16_ | Flavonol glycoside |
| Afzelin | C_21_H_20_O_10_ | Flavonol glycoside |
| Hyperoside | C_21_H_20_O_12_ | Flavonol glycoside |
| Avicularin | C_20_H_18_O_11_ | Flavonol glycoside |
| Reynoutrin | C_20_H_18_O_11_ | Flavonol glycoside |
| Rutin | C_27_H_30_O_16_ | Flavonol glycoside |
| Quercitrin | C_21_H_20_O_11_ | Flavonol glycoside |
| Quercetin-3β-D-glucoside | C_21_H_20_O_12_ | Flavonol glycoside |
| Catechin | C_15_H_14_O_6_ | Flavan-3-ol |
| (–)-Epicatechin | C_15_H_14_O_6_ | Flavan-3-ol |
| Procyanidin B1 | C_30_H_26_O_12_ | Proanthocyanidin (dimer) |
| Procyanidin B2 | C_30_H_26_O_12_ | Proanthocyanidin (dimer) |
| Procyanidin C1 | C_45_H_38_O_18_ | Proanthocyanidin (trimer) |
| Phloretin | C_15_H_14_O_5_ | Dihydrochalcone |
| Phlorizin | C_21_H_24_O_10_ | Dihydrochalcone glycoside |
| Trilobatin | C_21_H_24_O_10_ | Dihydrochalcone glycoside |
| Phloretin 2-O-xyloglucoside | C_26_H_32_O_14_ | Dihydrochalcone glycoside |

**Table S1** Polyphenols identified in FHLS by LC-HRMS analysis
